# The Problem of the Very Sick Citizen

**Published:** 1911-12-09

**Authors:** 


*To the Editor of* The Hospital.

### THE PROBLEM OF THE VERY SICK CITIZEN.

*To the Editor of* The Hospital.

Sir,—Every man, in a civilised State, has a right to live. If he has a right to live he has also a right to be kept alive. This necessitates skilled treatment—sometimes practicable only in hospitals.

The State therefore owes it to the citizen that hospitals for his treatment, when sick, shall be available when necessary. If private benevolence is prepared to supply this need—well and good. If not, the State must step in.

To some extent the State recognises its duty. If you are destitute, there is the workhouse. If you are destitute and sick, there is the workhouse infirmary.

But what if you are not destitute but only seriously ill? What if your trouble is not poverty but peritonitis, not destitution but disease—not abject want but appendicitis? If the hospitals supported by the charitable are full, what must you do? Has the State no duty towards you? Must you die at home?

How can the State, or the municipality, best discharge this obligation towards the very sick citizen? By providing hospitals? If necessary, preferably I think, by aiding private benevolence.

Those of us with any practical acquaintance with corporations or Poor Law boards are not greatly enamoured

